# Epistasis detectably alters correlations between genomic sites in a narrow parameter window

**DOI:** 10.1371/journal.pone.0214036

**Published:** 2019-05-31

**Authors:** Gabriele Pedruzzi, Igor M. Rouzine

**Affiliations:** Sorbonne Université, Institute de Biologie Paris-Seine, Laboratoire de Biologie Computationelle et Quantitative, LCQB, Paris, France; Illumina Inc, UNITED STATES

## Abstract

Different genomic sites evolve inter-dependently due to the combined action of epistasis, defined as a non-multiplicative contribution of alleles at different loci to genome fitness, and the physical linkage of different loci in genome. Both epistasis and linkage, partially compensated by recombination, cause correlations between allele frequencies at the loci (linkage disequilibrium, LD). The interaction and competition between epistasis and linkage are not fully understood, nor is their relative sensitivity to recombination. Modeling an adapting population in the presence of random mutation, natural selection, pairwise epistasis, and random genetic drift, we compare the contributions of epistasis and linkage. For this end, we use a panel of haplotype-based measures of LD and their various combinations calculated for epistatic and non-epistatic pairs separately. We compute the optimal percentages of detected and false positive pairs in a one-time sample of a population of moderate size. We demonstrate that true interacting pairs can be told apart in a sufficiently short genome within a narrow window of time and parameters. Outside of this parameter region, unless the population is extremely large, shared ancestry of individual sequences generates pervasive stochastic LD for non-interacting pairs masking true epistatic associations. In the presence of sufficiently strong recombination, linkage effects decrease faster than those of epistasis, and the detection of epistasis improves. We demonstrate that the epistasis component of locus association can be isolated, at a single time point, by averaging haplotype frequencies over multiple independent populations. These results demonstrate the existence of fundamental restrictions on the protocols for detecting true interactions in DNA sequence sets.

## Introduction

Epistasis is inter-dependence of the fitness effects of mutations occurring at different loci. The term ‘epistasis’ in population genetics refers to the fact that mutations occurring at different genomic sites affect the Darwinian fitness of an organism, i.e., its average progeny number, in a non-multiplicative fashion. At the cell biology level, this phenomenon is caused by various biological interactions [1–4]. In biological systems, amino acids in proteins domains interact with each other. The resulting networks of interactions that include direct protein-protein binding and allosteric effects, shape the gene regulation and metabolic networks. Epistasis is a widespread property of biological networks [2, 5–8] and a subject of intense studies. The vital role it plays in the genetic evolution of populations and the heritability of complex traits is well established. The existing estimates indicate that the variation of an inherited trait across a population can only partially be explained by the additive contributions from the relevant alleles. On average, 70% of the inheritance may be due to epistasis or epigenetic effects [9]. Epistasis defines the evolutionary paths and creates fitness valleys, i.e., intermediate genetic variants with reduced fitness [10–12].

A crucial biological scenario is a viral population adapting to the abrupt changes in external conditions. Examples include the transmission to a new host, the invasion of a new organ, or the process of immune evasion or the development of drug resistance. Typically, virus adaptation consists of primary mutations followed by a cascade of several compensatory (helper) mutations [13–18]. These mutations help the adapting virus to pass through a fitness valley [11]. During this process, compensatory mutations rescue the replicative fitness of virus while preserving its resistant phenotype [13, 15, 19].

However, epistasis is not the only force causing inter-dependence in the evolution of genomic regions. The other dominant factor is the host of linkage effects due to the fact that different loci in the absence of recombination (or under limited recombination) are linked, i.e., inherited together, as a set [20, 21]. The consequences of linkage include Fisher-Muller effect (clonal interference), genetic hitchhiking and genetic background effects, and Hill-Robertson interference between genetic drift and selection [21–23]. The effects of linkage on evolution in the presence of selection is well understood theoretically [12, 24–31]. The theory shows that linkage significantly slows adaptation, enhances accumulation of deleterious mutations, and changes the shape of the phylogenetic tree [32, 33]. The magnitude of linkage effects grows rapidly with the number of loci, *L*. Recombination partly offsets linkage effects and accelerates evolution [34–40] and competes with epistasis [41]. Epistasis has been shown to be potentially important for the evolution of recombination in a two-locus model [42, 43].

Another consequence of linkage, which represents the focus of the present work, is the strong interaction between the evolutionary trajectories of different sites. LD stemming from linkage is easy to confuse with epistasis effects. Linkage effects are stochastic, due to stochastic sampling of genomes and random nature of mutations. They become small only in populations that are exponentially large in the number of sites *L* [25]. Working with sequence data from real populations, it is often unclear how to discriminate the effects of shared ancestry from those of epistasis, and which of the two evolutionary forces dominates in each case (for a comprehensive review, see [1, 44, 45]). Therefore, despite of a considerable theoretical and experimental effort, detecting epistasis from genomic data remains a challenge.

In the present work, we offer an evolutionary explanation for the observed difficulty of the detection of epistasis from one-time data set. The idea is to generate mock data using a Monte-Carlo model of evolution and then try to discriminate between effects of linkage and epistasis. We use a panel of six pairwise LD measures to compare their distributions between epistatic and random pairs in a broad range of model parameters. We also use 3D and 2D maps of all possible combinations of LD measures and employ an optimization algorithm based on *a priori* knowledge to estimate the best, theoretically possible identification of epistatic pairs. As a result, we delineate the region of time and model parameters where the epistatic pairs can be detected against the linkage background. Finally, we investigate the role of recombination and the effects of averaging over multiple independently-evolving populations.

## Results

### Computer simulation of evolution

We consider a haploid population of *N* genomic sequences comprised of *L* sites, where *L* >> 1, and either a favorable or deleterious allele is present at each site. Evolution of the population between discrete generations is simulated using a Wright-Fisher model including the evolutionary factors of random mutation with the rate *μ* per site, random genetic drift, and natural selection, as described in *Methods*. Natural selection includes positive (antagonistic) epistatic interaction between selected pairs of deleterious alleles. A simple case of genomes with uniform selection coefficient *s*_0_ and uniform epistatic strength, *E*, is considered. We also assume that epistatic pairs are isolated, i.e., that each genomic site interacts with only one site. The initial population is randomized as it is done in virus passage experiments, with an average allelic frequency *f*_0_. In most of our work, we initially neglect the factor of recombination and primarily focus on asexual evolution, but lift this restriction in the end and explore broad parameter ranges. We aim to simulate the detection of epistatic pairs and identify the best conditions for detection theoretically.

### Measures of linkage disequilibrium (LD)

Various haplotype-based measures based on known haplotype frequencies have been proposed to characterize the allelic association between loci. We will list four measures, as follows.

Lewontin’s measure of statistical correlation between alleles at different loci has a form [46]

$$D^{\prime }=\frac{D}{D_{max}},{D=f}_{ij}-f_{i\;}f_{j}$$ (1)

Dmax={max⁡{-fifj,-(1-fi)(1-fj)},D<0min{fi(1-fj),(1-fi)fj},D>0

Here *f*_*ij*_ is the average frequency of a bi-allelic haplotype of loci *i* and *j*, and *D*_*max*_ is a normalization coefficient making sure that *D*′ ∈ [0, 1].

An alternative measure is Pearson correlation coefficient between pairs of loci *r*, expressed as [47]

$$r=D/\sqrt{f_{i}(1-f_{i})f_{j}(1-f_{j})}$$ (2)

More recently, Wu and colleagues [48] have proposed another statistical marker of linkage disequilibrium which has the bi-allelic form

$$WU=log\frac{f_{11}f_{00}}{{f_{01}f}_{10}}$$ (3)

which represents the logarithm of the *Z*-measure proposed previously by Kimura [49].

In our recent work [50], we introduced another bi-allelic measure

$$UFE=1-\frac{log(f_{11}/f_{00})}{log(f_{01}f_{10}/{f_{00}}^{2})}$$ (4)

The advantage of this measure with respect to previous three is that it has a direct meaning in terms of fitness. For isolated interacting pairs, when frequencies in Eq 4 are ensemble-averaged, it represents the degree of mutual compensation of two deleterious mutations, UFE = *E* (see Methods below). Here the value *E* = 0 corresponds to the absence of compensation, and *E* = 1 to full mutual compensation of the two mutations. We checked that the singularity in Eq 4 at *f*_10_ *f*_01_ = *f*_00_^2^ does not affect our results.

Below we investigate the effect of linkage for interacting and noninteracting pairs of loci using the measures defined in Eqs 1–4. Also, we employ an optimization algorithm that, exploiting a priori knowledge of the correct epistatic pairs, puts the best possible threshold between the two distributions of LD. We consider different combinations of two or three LD measures to obtain the best detection possible.

### LD of epistatic and non-epistatic pairs are distinct in a narrow parameter window

**Fig 1:**
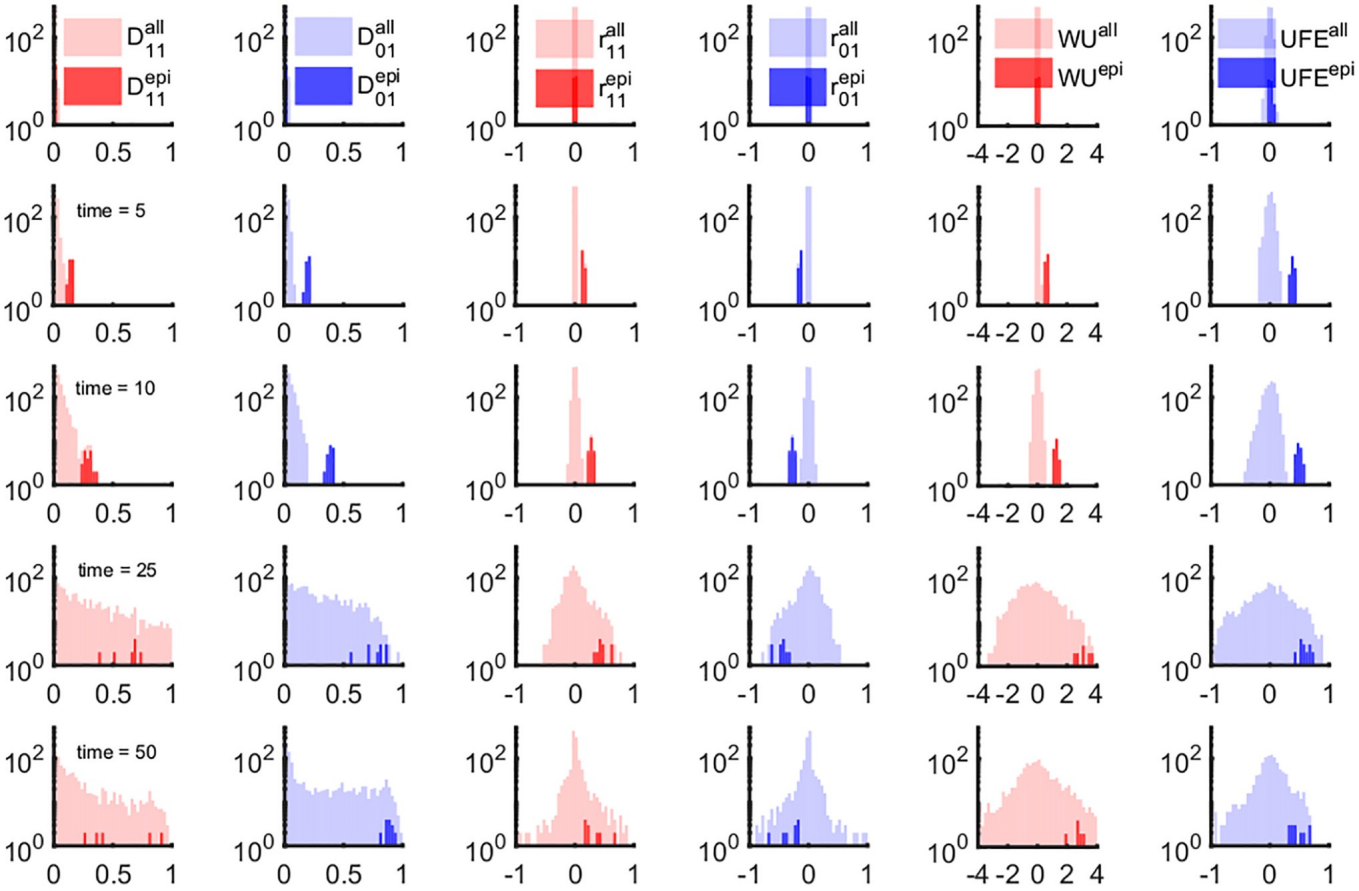
LD- and haplotype-based measures of epistasis identify a narrow time window of epistasis detectability. We compared the time-dependent distribution of 6 markers of LD shown in 6 columns. Each column shows the profile of the distribution of a measure of epistasis: *D'*_11_, *D'*_01_ (Eq 1), *r*_11_, *r*_01_ (Eq 2), WU (Eq 3) and UFE (Eq 4). Different rows correspond to different time points: *t* = 1, *t* = 5, *t* = 10, *t* = 25 and *t* = 50. The shaded regions correspond to the density distributions for all possible pairwise interactions (lighter color) and the known epistatic pairs (darker shade). The shaded areas are normalized distributions reflecting the fact that epistatic pairs represent a tiny fraction of the all possible pairs in a genome. The fluctuations of non-epistatic pairs increasing in time overlap onto the distributions of epistatic pairs. Parameters: *N* = 2 10^4^, *s*_0_ = 0.1, *L* = 50, *E* in the range [0, 1], *μL* = 7 10^−2^. Each odd site interacts with its neighbor on the right (1–2, 3–4, 5–6, …) with epistatic strength *E* = 0.75. Initially, sequences were random with average allelic frequency set to *f* = 0.4. The negative control result in the absence of epistasis (*E* = 0) is presented on S1 Fig.

We started by plotting the distribution of six LD measures calculated from Eq 1 over individual pairs of sites, at different times (Fig 1). We show separately the distribution for two subsets of pairs: the known epistatic subset (dark shade) and all the pairs (light shade). In the beginning, LD is narrowly distributed around zero, for both epistatic and non-epistatic subsets (Fig 1, row 1).

Subsequent time points (Fig 1, rows 2 and 3) show progressive separation of the two distributions. In the course of further evolution (Fig 1, rows 4 and 5), the distribution of randomly-chosen pairs, which was initially narrow and concentrated near the origin *E* = 0, gradually expands and overlaps with the small epistatic distribution (Fig 1). This effect implies that non-epistatic pairs of sites, due to the stochastic nature of evolution, produce large LD of random sign. In this case, it is impossible to tell apart epistatic pairs from any of these measures of LD.

### Results are robust to the choice of an LD measure or their combination

**Fig 2:**
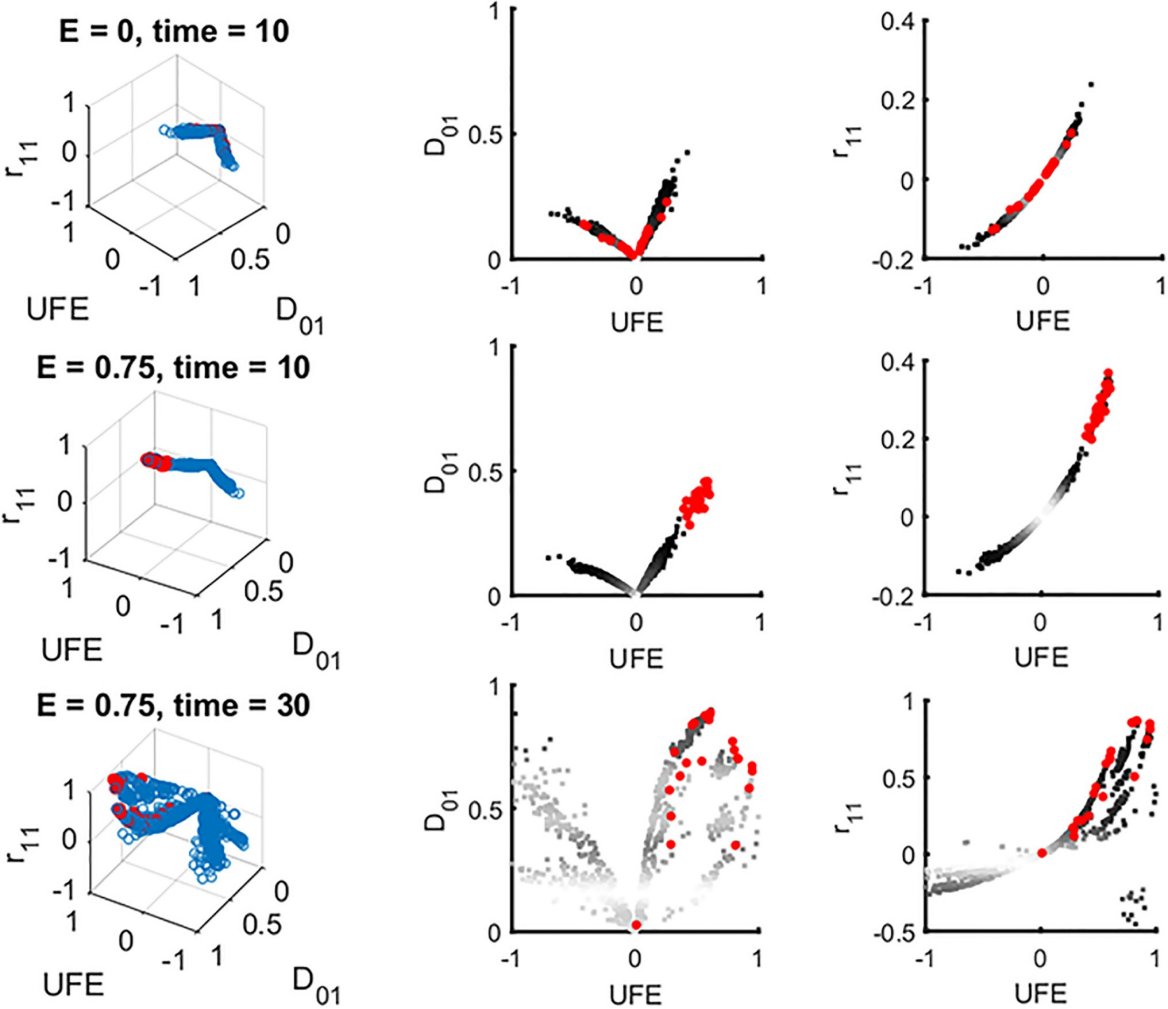
The optimization algorithm to identify ideal conditions for detection of epistasis is exemplified through the 3D scatter plot of three different measures of LD. Left: A representative example of three-dimensional scatter plots of three LD statistics, UFE, *D'*_01_ and *r*_11_, plotted for all pairs of sites (blue circles) and for designated epistatic pairs (red circles). Right and middle: two-dimensional projections. The upper row corresponds to zero epistasis (*E* = 0, top). Second and third row are two time points in the presence of epistasis, within and outside the detection window, respectively. All possible combinations of two and three measures have also been tested and summarized in S1 Table. At intermediate time *t* = 10, a distinct cloud of epistatic pairs (red dots) cluster separately from the other pairs and, hence, are detectable (middle row). At long times, substantial overlap with non-interacting pairs contaminates detection (bottom row). To optimize detection, we define a detection threshold for each statistics and use an optimization algorithm that minimizes the following quantity "DET + *a* FPOS", where *a* is a fitting parameter, DET represent the detection percentage, and FPSO is the percentage of false positive, based on prior knowledge of the identity of true epistatic pairs. Parameters are as in Fig 1.

Next, we checked whether combinations of LDs used together can improve detection. We have calculated all possible combination of six LD measures in Eq 2 and tried to separate interacting and non-interacting pairs using 3D and 2D scatter plots. A representative example is shown in Fig 2, for *E* = 0, and for *E* = 0.75 at two time points. Other possible combinations of 2 and 3 measures are summarized in S1 Table.

We wrote an optimization algorithm which separates the cloud of interacting pairs from the cloud of non-interacting pairs in the best possible way, using *a priori* knowledge about the identity of pairs (Fig 2). We adjusted the threshold to optimize the difference between the detection rare and the false positive rate. This method, employing the principle of machine learning, does not give any substantial improvement on the detection window (See S1 Table). For a real data sets, *a priori* knowledge about interacting pairs is usually unavailable, so that the detection of epistasis in a single population at one time point will be even worse than our prediction.

### Clonal exclusion has a minor effect on detection window

We also attempted to improve detection by analyzing the clone structure of population and excluding the largest clones from the simulated sequences set, which comprise a significant fraction of population [28] and could contribute to noise (S1 Appendix). We have reached only a slight expansion in the time window of detection (S2 and S3 Figs).

### Parameter sensitivity analysis confirms the narrow window of detection

#### Selection coefficient

**Fig 3:**
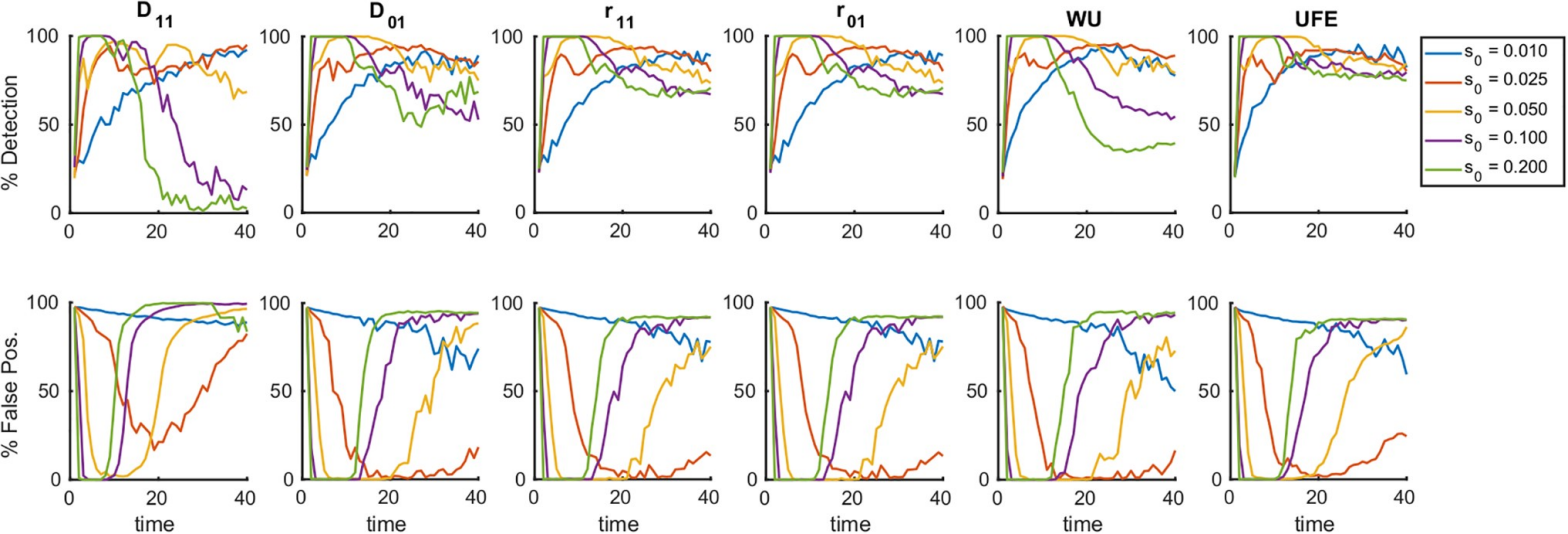
Detection of epistasis is confined in a time window whose width is controlled by the mean selection coefficient. Percentile of detection and false discovery as a function of time is averaged over 25 random simulation runs per each value of *s*_0_, the constant selection coefficient for each allele in the sub-population. For the average over 300 runs, see S4 Fig. The detection of epistatic pairs for a panel of measures of LD, namely, *D*_11_, *D*_01_ (Eq 1), *r*_11_, *r*_01_ (Eq 2), WU (Eq 3) and UFE (Eq 4). Results from a detection protocol that maximizes the difference between the detection percentile and the false-positive fractions by tuning the detection threshold, show the same trend for all measures considered. At time ~1.5/*s*_0_ generations, we observe the beginning of a transition which completely blurs the detection of epistatic interaction at time ~2.5/*s*_0_. The initial allelic frequency *f*_0_ = 0.45, *s*_0_ is shown, the other parameters are as in Fig 1.

Next, we investigated how the window of detection changes with model parameters. We calculated the detection rate and the false positive rate for the six measures of LD at different values of selection coefficient, *s*_0_ (Fig 3). For each measure, the results show an inverse scaling of the detection time window on *s*_0_. Note that the window closes at very small *s*_0_, where evolution is almost selectively neutral, and epistasis is never detectable.

#### Distributed selection coefficient

Next, we conducted a sensitivity analysis with respect to the other model parameters (S5 Fig). Firstly, we lifted the simplifying assumption of a constant selection coefficient, *s* = *s*_0_, and allowed variation of *s* among sites according to a half-Gaussian distribution. We obtain a similar dependence of the window width on the average selection coefficient (S5 Fig), although with a higher false positive rate within the detection window than for the case with constant *s*.

#### Length of the genome

We found out, that sequence length *L* limits the detectability of epistasis substantially (S5 Fig). An increase of the sequence length or a reduction of the population size leads to narrowing and, eventually, disappearance of the detection window. These results limit the applicability of these methods to short sequences. Indeed, the number of all possible locus pairs increases with genome length *L* proportionally to *L*^2^, and the number of epistatic pairs increases only as *L*, so that the task of finding "the ruby in the rubbish" becomes harder at larger *L* [1, 44, 45].

#### Population size

We observed a very slow (logarithmic) expansion of the detection window with population size *N* (S5 Fig). This is consistent with the results of asexual evolution models, which predict a very slow logarithmic dependence on *N* for all the evolutionary observables, including evolution speed, genetic diversity, and the average time to most recent ancestor [25–31, 35–37, 39, 40, 51]. Only in very large populations whose size increases exponentially genome length *L*, linkage effects become small [25]. In these, astronomically large populations, epistasis would be easily detectable.

#### Initial standing variation

We have observed a detection window in time only at the initial frequencies of deleterious alleles above 10% (S5 Fig). At smaller frequencies, detection lapses. We can conclude that detection of epistasis in a single population studied is possible in a narrow parameter range.

### Recombination improves detection

**Fig 4:**
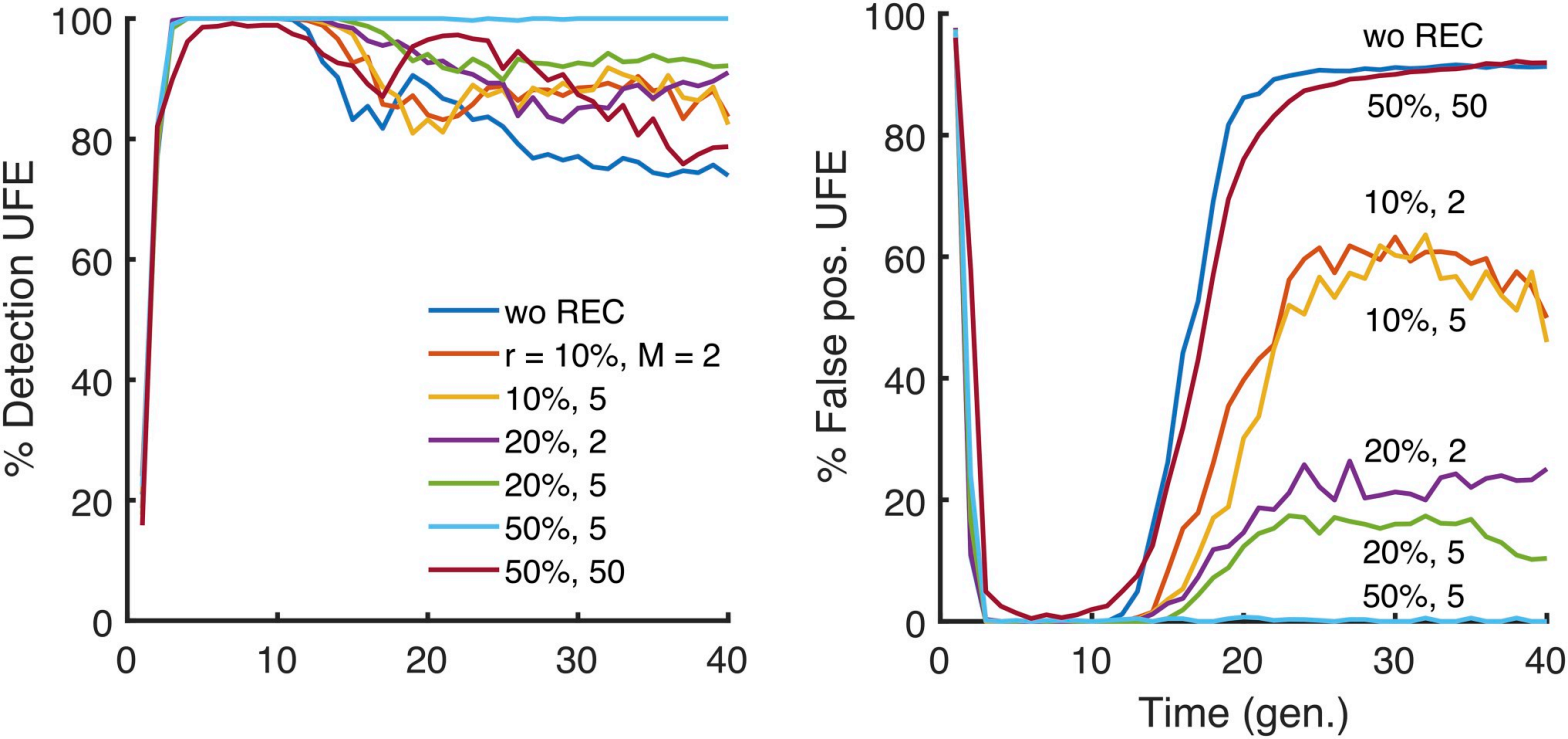
Variation of the time window of detection with recombination. Percentile of detection and false discovery as a function of time is averaged over 25 random simulations (runs) in a broad range of parameters values. The detection rare and false positive rate of epistatic pairs with UFE at different values of *s*, randomly drawn from a half-Gaussian distribution of deleterious alleles. The presence of moderate recombination characterized by outcrossing rate *r* and the average number of cross-overs, *M*, broadens the detection window. We observe similar results for all the statistics considered in this study (S5–S10 Figs). The default parameter set is *E* = 0.75, with the other parameters as in Fig 1.

Until now, we have assumed a completely asexual evolution. In our next step, we investigated the role of recombination, parametrised by the average number of crossovers per genome, *M*, and by the probability of outcrossing per genome, *r*. We obtained that intermediate recombination rates rescue the detection of epistasis by disrupting linkage and yet preserving the epistasis contribution to LD. At our default parameter set (Fig 1 legend), we observed a significant reduction of linkage fluctuations starting from *r* = 20% and *M* = 5 (Fig 4). The results show that LD effects of linkage are much more resistant to recombination than, for example, the evolution speed, which increases substantially already at tiny values of *r* [34–40]. We found out also that extremely high levels of recombination decrease LD for epistatic pairs as well, thus rendering epistasis undetectable. Thus, there exists a narrow window of recombination rates where epistasis can be observed outside of the detection window for time and other parameters described above.

### Population divergence creates strong linkage effects

**Fig 5:**
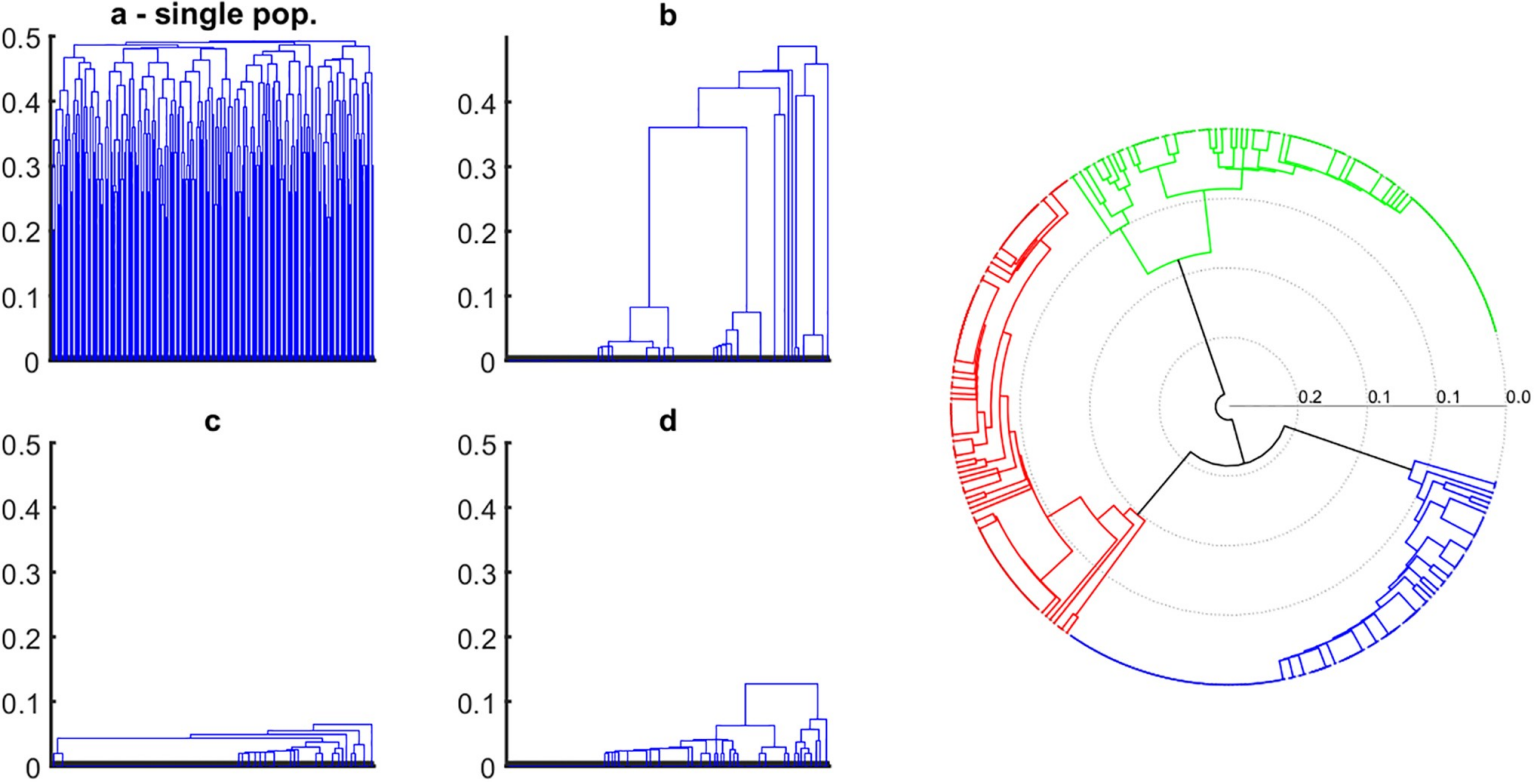
Evolution of genealogy within a single, well-mixed population and comparative representation of multiple, independently evolving population. (a-d) Phylogenetic structure of a single population comprising a sample of 500 genomes at four different times: *t* = 0, 10, 20, and 30 generations. Mean genetic distance between genomes decreases in time, and the structure of the tree changes from a star-like shape towards a monophyletic tree (BS coalescent), with a single common ancestor. The right panel shows the reconstructed phylogenetic tree of three populations, independently evolved from the same initial random seed. At a glance, it is possible to determine that the three populations do not share much sequence homology and segregate into different, phylogenetically distinct clades. *N* = 20000 genomes, initial average allelic frequency *f*_0_ = 0.40, other parameters as in Fig 1.

In order to understand the reason behind the strong linkage effects masking epistasis, we investigated the time-dependent changes of the phylogenetic tree using a hierarchical clustering algorithm (Fig 5a–5d). The initial, randomized population display a star-shaped phylogeny, characterized by the same mean distance between all sequences and the most common sequence (Fig 5). With time, the phylogenetic tree grows branches of increasingly related sequences (Fig 5c and 5d). As simulation continues (Fig 5d), the tree becomes more lopsided, while recent mutations create short branches at the bottom. At the same time, we observe that the tree has a decreasing number of ancestors. Eventually, the tree evolves into Bolthausen-Sznitman coalescent (BSC) with a single common ancestor, previously predicted for the stationary regime of traveling wave [25, 29, 37] (Fig 5).

Emergence of this phylogeny is coincident with the increase in the fluctuations of LD of non interacting pairs (Fig 1). The reason for strong random LD is stochastic divergence of the population from the initial state, as illustrated by clustering of three independently evolved populations (Fig 5, right). The distance between the trees obtained in separate runs increases linearly in time due to fixed beneficial mutations at randomly chosen sites. Haplotype configurations of the common ancestor of the population are inherited by all members of the population, with some small variation determined by the time to the most recent common ancestor. Thus, the stochastic divergence of individual populations creates strong LD with a random sign.

### The use of multiple populations defeats LD fluctuations and rescues epistatic signature

**Fig 6:**
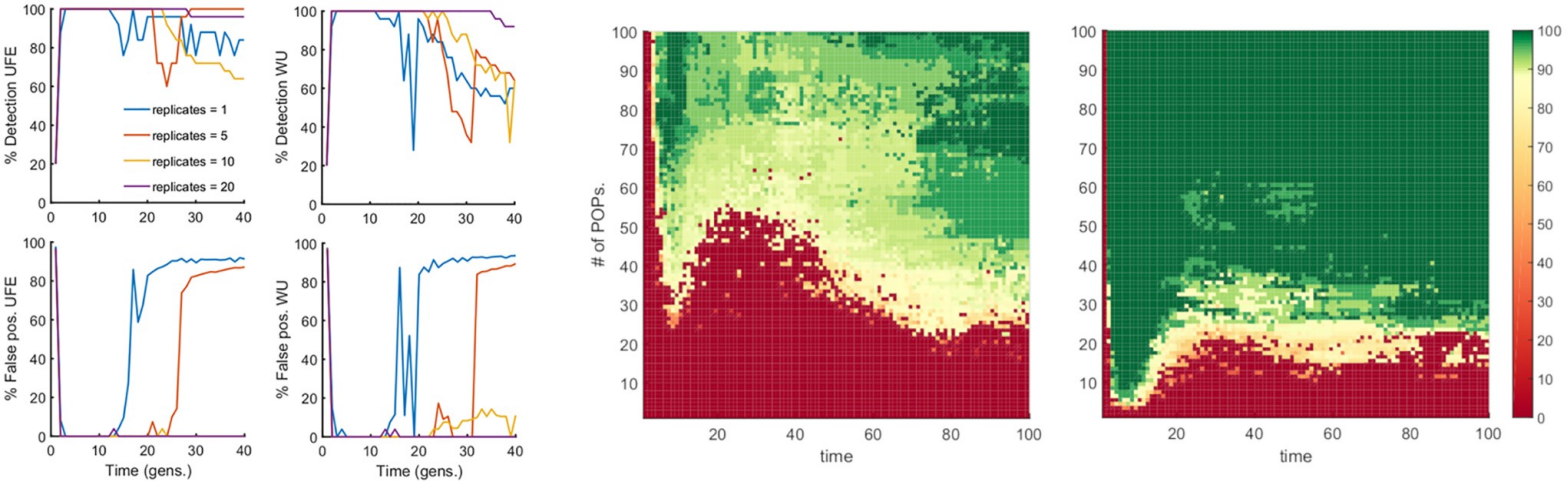
Detection of epistasis is rescued by simultaneous analysis of multiple independently-evolved populations. Left 4 plots: Percentile of detection (top) and false discovery (bottom) as a function of time are presented for UFE and WU measures. Number of replicate Monte-Carlo runs is shown. The haplotype frequencies are averaged over runs, which represent independently-evolved populations. At time ~1.5/*s*_0_, we observe the beginning of a transition which completely blurs the detection of epistatic interaction for a single replicate (blue line), however, already 5 replicates are sufficient to significantly extend the detection window up to ~2.5/*s*_0_, and a higher number of replicates completely eliminate false-positive pairs, while maintaining the average detection above 80%. Parameters: *E* = 0.75, *N* = 20000, the others as in Fig 1. Right: Two-dimensional color maps for UFE measure of LD, which summarize the results of a similar analysis for two population sizes: *N* = 100 (middle plot) and *N* = 1000 (right plot). *Y*-axis: Number of independent populations. *X*-axis: time of evolution. Color shows the percentage of detection with the detection threshold of interacting pairs chosen to give the false discovery rate below 20%.

Because the linkage fluctuations arise due to stochastic divergence of the founder, the common ancestor, the natural idea is to use multiple populations to average over possible founder sequences. To test this idea, we evolved independently multiple populations at the same initial conditions and averaged the haplotype frequencies used in LD markers (Eqs. 1–4) over populations, for each pair of sites, separately. We found out that including a sufficient number of independent populations results in a substantial reduction of the noise and indefinite expansion of the window of detection (Fig 6). Qualitatively similar results are obtained for all LD markers.

## Discussion

In the present work, using a Monte-Carlo simulation of a haploid population, we calculated the distributions of six measures of linkage disequilibrium and their combinations for epistatic and random locus pairs. We demonstrated that, in a single asexual population, the footprints of epistatic pairs are readable only in a narrow time interval between 0.2/*s*_0_ and 1.5/*s*_0_ generations. During later adaptation, the distribution of linkage disequilibrium for non-interacting pairs broadens and engulfs the distribution for epistatic pairs. These results indicate that, long before the onset of the steady state, linkage effects dominate over the effects of epistasis. This phenomenon is predicted in a broad parameter region and for all the LD statistics, suggesting that, in the context of inherited linkage fluctuations, all statistics based on pairwise linkage disequilibrium are equal.

To gain insight into the evolutionary origin of these fluctuations, we investigated phylogenetic trees of the entire population at different time points to observe that the shape of the tree strongly correlates with the magnitude of linkage fluctuations. The shape of the phylogenetic tree changes in time from the initially star-shaped genealogy to a Bolthausen-Sznitman (BS) coalescent [32, 33] previously analyzed in great detail for adapting asexual populations [25, 36, 37]. Once BS genealogy is established, individual sequences share a high degree of interrelatedness due to fixed beneficial mutations at randomly chosen sites. The presence of the BS coalescent is coincident with strong co-inheritance linkage fluctuations. The stochastic nature of their common ancestor sequence, divergent in time from common ancestors in other independent populations (Fig 5) is the cause of the strong fluctuations of LD.

We have also directly quantitated the detection of epistatic pairs against the background of random linkage effects. We evaluated the sensitivity of the width of the detection window with respect to several input parameters, such as the mean selection coefficient, the size of the population, the sequence length, and initial genetic variation, and the role of recombination. We observed that the window is proportional to the inverse average selection coefficient, 1/*s*_0_, but a very small *s*_0_ abolishes any chance of detection, so that the best detection is attained in the case of moderately weak selection. The detection window exists only for sufficiently small genomes. The presence of recombination has the effect of compensating the linkage component and thus significantly improving the detection of epistasis. Yet, very frequent recombination disrupts epistatic effects.

To isolate the epistatic component from co-inheritance effects, we performed simulations over several independently-evolved populations and averaged the haplotype frequencies over these runs. The results predict the number of independent population required to attain significant expansion of the detection window (Fig 6). Thus, the averaging over multiple independently-evolved populations filters out linkage effects leaving a clear footprint of epistasis in a much broader parameter range. However one should note that the multiple-population sampling was conducted under the ideal conditions, in which every population evolved independently for the same time with the same parameter set, and represented the same fraction of the total sample. Unequal sampling or heterogeneous representation in real data sets may create additional problems.

Our model adopts several simplifying assumptions. (i) Deleterious alleles are assigned selection coefficient constant in time. (ii) We considered constant and fixed epistatic strength for all pairs. (iii) We focused on a simple topology of epistatic network. While these are reasonable assumptions to describe the problem of linkage fluctuations in biological systems, a real scenario with mixed sign epistasis and complex topology might pose additional challenges for the accurate detection of epistasis.

The results obtained from averaging over independent populations give strong evidence for the role of stochastic divergence in linkage statistics. There are some cases, such as virus evolution in independent populations where it is possible to obtain independent replicates. Examples include influenza virus sampled in different countries, virus passage in parallel tissue cultures, cancer cell evolution in different organs. For the study of human genetics, it may be possible to obtain independent isolates from under-mixed subpopulations that split long time ago. In principle, one can try to use data from different countries of common origin. For example, full genome studies show that European nations, despite of interbreeding and outbreeding, remain genetically distinct after a split from a common origin ~6000 years ago (see Fig 2 in [52]). Therefore, they can be viewed as quasi-independent populations with weak genetic exchange. Our results imply that comparing genetic data from related but distinct ethnicities allows to study epistasis more reliably than in a single ethnic group.

## Conclusions

We identified the evolutionary reason for strong fluctuations of epistatic estimates in the existing sequence sets. Linkage due to stochastic divergence of the common ancestor of a population from the origin is responsible for the high false-positive rates of epistasis detection in a single population. We demonstrated how the use of multiple independently-evolving populations allows to average out strong linkage effects and rescue the detectability of epistasis.

## Materials and methods

We consider a haploid population of *N* binary sequences, where each genome site (nucleotide position) numbered by *i* = 1, 2, …, *L* is either *K*_*i*_ = 0 or *K*_*i*_ = 1. We assume that the genome is long, *L* >> 1. Evolution of the population in discrete time measured in generations is simulated using a standard Wright-Fisher model, which includes the factors of random mutation with rate *μL* per genome, natural selection, and random genetic drift. Recombination is assumed to be absent. Once per generation, each genome is replaced by a random number of its progeny which obeys multinomial distribution. The total population stays constant with the use of the broken-stick algorithm.

To include natural selection, we calculate fitness (average progeny number) *e*^*W*^ of sequence *K*_*i*_ as given by [50]

$$W=\sum_{i=1}^{L}s_{i}K_{i}+\sum_{i<j}^{L}S_{ij}K_{i}K_{j}$$ (5)

$$S_{ij}=E_{ij}(\left|s_{i}|+|s_{j}\right|)T_{ij}$$ (6)

The biological meaning of this expression is, as follows. According to a well-know theorem of population genetics, different loci are predicted to evolve independently in a large population if contributions of mutations occurring at different loci to organism’s log fitness are additive, which corresponds to the case of biologically non-interacting sites. Formally, this situation is described by the first term in Eq 5 with additive contribution of single mutations to fitness, with selection coefficient *s*_*i*_ for each site *i*. Interaction between loci creates non-additive effects to the fitness log: the second term in Eq 5 describes pairwise interactions of sites with magnitudes *S*_*ij*_ given by Eq 6.

Coefficient *E*_*ij*_ introduced in Eq 6 represents the relative strength of epistatic interaction between sites *i* and *j*, while the binary elements of matrix **T** indicate the interacting pairs by *T*_*ij*_ = 1 and the other pairs by *T*_*ij*_ = 0. An example of positive epistasis is the compensation of two deleterious mutations inside protein segments that bind each other. Note that *E*_*ij*_ = 1 corresponds to full mutual compensation of deleterious mutants at sites *i* and *j*. We consider the simplest interaction topology of interacting neighbors, as given by *T*_2*i*,2*i*+1_ = 1 and 0 for all other pairs.

Here we include only pairwise interactions, neglecting higher-order interactions between protein residues. Even though non-pairwise models are sometimes used in the literature, we are not aware of any evidence that higher-order interactions are significant in viruses or any other organisms.

## Supporting information

S1 Table Theoretical limits of detection of epistasis, expressed as the percentages of detection and false positives. We devised an optimization algorithm that, based on prior knowledge of the true epistatic association, identifies threshold within the data that allow to sort epistatic pairs from non-interacting ones. We present data for each induvial estimator of epistasis and for combinations of two, and three measure simultaneously. The analysis was repeated at two time points (*T*_1_ = 10 and *T*_2_ = 30 generations, within and outside of the widows of detection, respectively. The results offer a comparative perspective over the detection performances of different measure and show that all LD- and haplotype-based estimators of epistasis can only detect true association, with reduced error and bias, only at *T*_1_, while at the later time point the magnitude of CI effects mask the epistatic associations.
(PDF)

S1 Appendix Clonal exclusion does not remove the limit to the detection of epistasis in a single-population. (PDF)

S1 Fig The time-dependent profile of the distributions of UFE and WU in the absence of epistasis (*E* = 0). The shaded areas are the distribution density of UFE (Eq 4, upper row, red) or WU (Eq 3, bottom row, blue). All possible pairwise interactions are shown by light shades, versus the distribution of epistatic pairs known *a priori* (dark shades). Between times of 0 and 50, the set of 20,000 sequences with a high standing variation evolves in the presence of the factors of mutation, selection, linkage, and random drift into broader (plus larges) distributions due to the co-inheritance linkage. Both the epistatic and all pair distributions are centered at the origin and overlap. This setting serves as a negative control to Fig 2 and confirms that the noise of UFE is an inherent structural component of the system and independent of the presence of epistasis. Parameters: *N* = 2 10^4^, *s*_0_ = 0.1, *L* = 50, *E* = 0, *μL* = 7 10^−2^. Each odd site interacts with its neighbor to the right (1–2, 3–4, …). Initially, sequences are randomized with the average deleterious allelic frequency set to *f* = 0.4.
(TIFF)

S2 Fig Clonal structure within unequal fitness classes emerges over time. Results show (a − e) the establishment of the clonal structure in discrete fitness classes, in the absence (*E* = 0, upper row) or in the presence of epistasis (*E* = 0.75, lower row) for a large asexual population. Stacked boxes represent the size of clonal lineages within each fitness class. The population is initially highly diverse (is composed of *N* unique clones), new mutations create a subpopulation, which drifts randomly at the higher end of the fitness distribution, meanwhile the less fit clones are selected out, and the fitness distribution travels rightward. Parameters: *N* = 2 10^4^, *s*_0_ = 0.1, *L* = 50, *μL* = 7 10^−2^. Each odd site interacts with its neighbor to the right (1–2, 3–4, etc.). Initially, sequences were random with allelic frequency set to *f* = 0.4.
(TIFF)

S3 Fig Clonal exclusion reduces the noise in a time-dependent manner. X-axis: the number of excluded clones. Y-axis: the variance-to-mean ratio, which can be considered as a measure of fluctuation amplitude. The dotted blue lines track the percentage of excluded sequences. The black dotted lines show the predicted decrease of noise assuming that all clones are statistically independent. Parameter values are as in Fig 1 with *E* = 0.75. These results show the decrease of the variance-to-mean ratio of the distribution of haplotype frequencies over time.
(TIFF)

S4 Fig Detection of epistasis is restrained to a limited time window. Percentile of detection and false discovery as a function of time is averaged over 300 random simulation runs. The detection of epistatic pairs for two measures, UFE (Eq 4) and WU (Eq 3), follows an optimization protocol that minimizes the sum of detection and false positive fractions by tuning the detection threshold set based on distribution of random sample of pairs (see the details in S3 Fig). At time ~1.5/*s*_0_, we observe the beginning of a transition which completely blurs the detection of epistatic interaction at time ~2.5/*s*_0_, coincident with the establishment of BS coalescent (Fig 5). These results describe a possible rationale for the design of an experimental setting that could detect epistatic interactions. Parameters: *N* = 2 10^4^, *s*_0_ = 0.1, *E* = 0.75, *L* = 50, *μL* = 7 10^−2^. Each odd site interacts with its neighbor to the right (1–2, 3–4, etc.). Initially, sequences were random with allelic frequency set to *f* = 0.4.
(TIFF)

S5 Fig Sensitivity analysis of epistasis detection with respect to model parameters for UFE measure of linkage disequilibrium (Eq 4). Percentile of detection and false discovery as a function of time is averaged over 25 random simulations (runs) in a broad range of parameters values. The default parameter set is *E* = 0.75, with the other parameters as in Fig 1. Parameters values are shown. Thick lines correspond to default parameter values used in Fig 1. (a) Distributed values of *s*, randomly drawn from a half-Gaussian distribution of deleterious alleles with average *s*_0_ (shown). (b) Increasing genome length *L* closes the detection window. (c) Decrease in population size *N* narrows the detection window. (d) The initial standing variation with average frequency of deleterious alleles *f*_0_ allows detection in a narrow time window at *f*_0_ > 10%.
(TIF)

S6 Fig Sensitivity analysis of epistasis detection with respect to model parameters for WU measure of linkage disequilibrium (Eq 3). Notation as in S5 Fig.
(TIF)

S7 Fig Sensitivity analysis of epistasis detection with respect to model parameters for Lewontin *D*_11_' measure of linkage disequilibrium (Eq 1). Notation as in S5 Fig.
(TIF)

S8 Fig Sensitivity analysis of epistasis detection with respect to model parameters for Lewontin *D*_01_' measure of linkage disequilibrium (Eq 1). Notation as in S5 Fig.
(TIF)

S9 Fig Sensitivity analysis of epistasis detection with respect to model parameters for Pearson coefficient *r*_11_ as a measure of linkage disequilibrium (Eq 2). Notation as in S5 Fig.
(TIF)

S10 Fig Sensitivity analysis of epistasis detection with respect to model parameters for Pearson coefficient *r*_01_ as a measure of linkage disequilibrium (Eq 2). Notation as in S5 Fig.
(TIF)
