# Supplementary material for: Epistasis detectably alters correlations between genomic sites in a narrow parameter window
Source: PLoS One. 2019 May 31;14(5):e0214036. doi: 10.1371/journal.pone.0214036 (PMC6544209; doi:10.1371/journal.pone.0214036)
Supplement: S1 Table — We devised an optimization algorithm that, based on prior knowledge of the true epistatic association, identifies threshold within the data that allow to sort epistatic pairs from non-interacting ones. We present data for each induvial estimator of epistasis and for combinations of two, and three measure simultaneously. The analysis was repeated at two time points (T1 = 10 and T2 = 30 generations, within and outside of the widows of detection, respectively. The results offer a comparative perspective over the detection performances of different measure and show that all LD- and haplotype-based estimators of epistasis can only detect true association, with reduced error and bias, only at T1, while at the later time point the magnitude of CI effects mask the epistatic associations. (PDF) [file pone.0214036.s001.pdf]

Table S1: Theoretical limits of detection of epistasis, expressed as the percentages of detection and false positives.

We devised an optimization algorithm that, based on prior knowledge of the true epistatic association, identifies threshold within the data that allow to sort epistatic pairs from non-interacting ones. We present data for each induvial estimator of epistasis and for combinations of two, and three measure simultaneously. The analysis was repeated at two time points (T_1 = 10 and T_2 = 30 generations, within and outside of the widows of detection, respectively. The results offer a comparative perspective over the detection performances of different measure and show that all LD- and haplotype-based estimators of epistasis can only detect true association, with reduced error and bias, only at T_1, while at the later time point the magnitude of CI effects mask the epistatic associations.

**Measures**			**DET_T1**	**FPOS_T1**	**DET_T2**	**FPOS_T2**
f_{00}			74,20	14,97	89,20	92,17
f_{01}			91,40	2,81	76,60	82,14
f_{10}			90,20	4,56	77,60	92,52
f_{11}			96,80	1,53	88,60	90,18
UFE			100,00	0,00	92,40	75,52
WU			100,00	0,00	92,20	73,70
D_{11}			94,00	28,84	51,00	97,13
D_{01}			100,00	0,00	87,00	79,98
r_{11}			100,00	0,19	91,40	71,42
D_{01}			100,00	0,19	91,40	71,42
f_{00}	*AND*	f_{01}	87,40	2,75	70,80	72,04
f_{00}	*AND*	f_{10}	84,40	4,85	69,00	82,38
f_{00}	*AND*	f_{11}	96,20	1,32	33,40	15,28
f_{00}	*AND*	UFE	76,40	12,53	71,40	56,47
f_{00}	*AND*	WU	75,60	12,61	82,60	69,72
f_{00}	*AND*	D_{11}	92,20	9,17	15,80	23,50
f_{00}	*AND*	D_{01}	77,20	13,61	70,00	48,02
f_{00}	*AND*	r_{11}	75,60	12,22	78,40	67,57
f_{00}	*AND*	D_{01}	75,60	12,22	78,40	67,57
f_{01}	*AND*	f_{10}	97,00	0,93	53,40	36,47
f_{01}	*AND*	f_{11}	97,80	0,58	64,00	55,55
f_{01}	*AND*	UFE	92,40	2,99	71,80	54,80
f_{01}	*AND*	WU	92,40	2,80	68,40	47,76
f_{01}	*AND*	D_{11}	98,00	3,68	24,60	36,52

f_{01}	*AND*	D_{01}	92,80	2,72	71,60	58,90
f_{01}	*AND*	r_{11}	92,80	2,72	74,00	59,73
f_{01}	*AND*	D_{01}	92,80	2,72	74,00	59,73
f_{10}	*AND*	f_{11}	96,40	1,42	66,00	65,22
f_{10}	*AND*	UFE	92,20	5,98	75,60	68,81
f_{10}	*AND*	WU	92,20	5,98	74,60	69,77
f_{10}	*AND*	D_{11}	97,80	5,87	32,80	51,04
f_{10}	*AND*	D_{01}	91,80	5,46	69,60	62,22
f_{10}	*AND*	r_{11}	92,20	6,01	74,00	66,23
f_{10}	*AND*	D_{01}	92,20	6,01	74,00	66,23
f_{11}	*AND*	UFE	97,60	1,97	81,80	75,80
f_{11}	*AND*	WU	97,40	1,57	81,60	73,11
f_{11}	*AND*	D_{11}	99,20	4,62	34,20	18,73
f_{11}	*AND*	D_{01}	97,80	1,90	73,80	57,98
f_{11}	*AND*	r_{11}	97,60	2,09	82,00	75,52
f_{11}	*AND*	D_{01}	97,60	2,09	82,00	75,52
UFE	*AND*	WU	100,00	0,00	91,00	70,50
UFE	*AND*	r_{11}	100,00	4,08	38,40	37,16
UFE	*AND*	D_{01}	100,00	0,00	83,00	62,33
UFE	*AND*	r_{11}	100,00	0,00	90,60	73,70
UFE	*AND*	D_{01}	100,00	0,00	90,60	73,70
WU	*AND*	D_{11}	100,00	4,08	32,80	2,83
WU	*AND*	D_{01}	100,00	0,00	83,60	63,81
WU	*AND*	r_{11}	100,00	0,00	90,20	68,89
WU	*AND*	D_{01}	100,00	0,00	90,20	68,89
D_{11}	*AND*	D_{01}	100,00	0,56	47,40	56,54
D_{11}	*AND*	r_{11}	100,00	4,08	33,00	2,83
D_{11}	*AND*	D_{01}	100,00	4,08	32,80	2,83
D_{01}	*AND*	r_{11}	100,00	0,00	87,00	69,57
D_{01}	*AND*	D_{01}	100,00	0,00	87,00	69,57
r_{11}	*AND*	D_{01}	100,00	0,00	92,80	75,02
f_{00}	*OR*	f_{01}	87,40	2,75	70,80	72,04
f_{00}	*OR*	f_{10}	84,40	4,85	69,00	82,38
f_{00}	*OR*	r_{11}	96,20	1,32	33,40	15,28
f_{00}	*OR*	UFE	76,40	12,53	71,40	56,47
f_{00}	*OR*	WU	75,60	12,61	82,60	69,72
f_{00}	*OR*	D_{11}	92,20	9,17	15,80	23,50
f_{00}	*OR*	D_{01}	77,20	13,61	70,00	48,02
f_{00}	*OR*	r_{11}	75,60	12,22	78,40	67,57
f_{00}	*OR*	D_{01}	75,60	12,22	78,40	67,57
f_{01}	*OR*	f_{10}	97,00	0,93	53,40	36,47

f_{01}	*OR*	r_{11}	97,80	0,58	64,00	55,55
f_{01}	*OR*	UFE	92,40	2,99	71,80	54,80
f_{01}	*OR*	WU	92,40	2,80	68,40	47,76
f_{01}	*OR*	D_{11}	98,00	3,68	24,60	36,52
f_{01}	*OR*	D_{01}	92,80	2,72	71,60	58,90
f_{01}	*OR*	r_{11}	92,80	2,72	74,00	59,73
f_{01}	*OR*	D_{01}	92,80	2,72	74,00	59,73
f_{10}	*OR*	r_{11}	96,40	1,42	66,00	65,22
f_{10}	*OR*	UFE	92,20	5,98	75,60	68,81
f_{10}	*OR*	WU	92,20	5,98	74,60	69,77
f_{10}	*OR*	D_{11}	97,80	5,87	32,80	51,04
f_{10}	*OR*	D_{01}	91,80	5,46	69,60	62,22
f_{10}	*OR*	r_{11}	92,20	6,01	74,00	66,23
f_{10}	*OR*	D_{01}	92,20	6,01	74,00	66,23
f_{11}	*OR*	UFE	97,60	1,97	81,80	75,80
f_{11}	*OR*	WU	97,40	1,57	81,60	73,11
f_{11}	*OR*	D_{11}	99,20	4,62	34,20	18,73
f_{11}	*OR*	D_{01}	97,80	1,90	73,80	57,98
f_{11}	*OR*	r_{11}	97,60	2,09	82,00	75,52
f_{11}	*OR*	D_{01}	97,60	2,09	82,00	75,52
UFE	*OR*	WU	100,00	0,00	91,00	70,50
UFE	*OR*	D_{11}	100,00	4,08	38,40	37,16
UFE	*OR*	D_{01}	100,00	0,00	83,00	62,33
UFE	*OR*	r_{11}	100,00	0,00	90,60	73,70
UFE	*OR*	D_{01}	100,00	0,00	90,60	73,70
WU	*OR*	D_{11}	100,00	4,08	32,80	2,83
WU	*OR*	D_{01}	100,00	0,00	83,60	63,81
WU	*OR*	r_{11}	100,00	0,00	90,20	68,89
WU	*OR*	D_{01}	100,00	0,00	90,20	68,89
D_{11}	*OR*	D_{01}	100,00	0,56	47,40	56,54
D_{11}	*OR*	r_{11}	100,00	4,08	33,00	2,83
D_{11}	*OR*	D_{01}	100,00	4,08	32,80	2,83
D_{01}	*OR*	r_{11}	100,00	0,00	87,00	69,57
D_{01}	*OR*	D_{01}	100,00	0,00	87,00	69,57
r_{11}	*OR*	D_{01}	100,00	0,00	92,80	75,02
