# Supplementary material for: Epistasis detectably alters correlations between genomic sites in a narrow parameter window
Source: PLoS One. 2019 May 31;14(5):e0214036. doi: 10.1371/journal.pone.0214036 (PMC6544209; doi:10.1371/journal.pone.0214036)
Supplement: S1 Appendix — (PDF) [file pone.0214036.s002.pdf]

S1 Appendix. Clonal exclusion does not remove the limit to the detection of epistasis in a single-population.

Aiming to reduce the overall noise present in the system, we went on to dissect the clonal structure of each fitness class, as follows. Before calculating haplotype frequencies in Eq. 4 for UFE measure of LD, we removed the first n most prominent clones from each fitness class. Then, for each haplotype frequency f_{00}, f_{01}, f_{10}, f_{11}, we calculated the relative noise magnitude as a function of the number of excluded clones (Fig. S3). To do that, we used the starting approximation that the clones are statistically independent and, hence, the variance of UFE is additive over clones. The contribution of each clone to noise depends on its size: the more significant the clone, the more it contributes.

As we expected, this method of clone exclusion produced significant noise reduction, but the magnitude of reduction strongly depends on time. In a narrow time window, it matches the analytic prediction derived from the approximation of statistically independent clones (Fig. S3, top right, dashed black line). At the time ~$1.5/s_0$, when 20 clones are excluded from the population, the noise decreases by 60% (Fig. S4, top right). At later time points, when the system enters into the stationary traveling wave regime, the clone exclusion becomes less and less efficient (Fig. S3, bottom row). These results indicate that the clonal exclusion can, in principle, reduce the noise in the system, but only in a narrow time window, and this result is typical for all measured haplotype frequencies. This is because the assumption of statistical independence of clones with sizes $1/i$ does not apply in the long run due to the common ancestry of clones.

All these findings are reflected in the false-positive rate and the detection power (Fig. S4). We predict excellent detection in the time window $(0.3\text{-}1.5)/s_0$ (see Fig. S3 for a detailed explanation of the detection algorithm). The exclusion of up to 5 most significant clones only slightly broadens this narrow window (Fig. S4). We conclude that the common origin of clones in the traveling-wave regime makes them statistically inter-dependent and restrain the detectability of epistasis to a narrow time window, which is not expanded by the procedure of clonal exclusion.
