# Supplementary figures and images for: Epistasis detectably alters correlations between genomic sites in a narrow parameter window
Source: PLoS One. 2019 May 31;14(5):e0214036. doi: 10.1371/journal.pone.0214036 (PMC6544209; doi:10.1371/journal.pone.0214036)

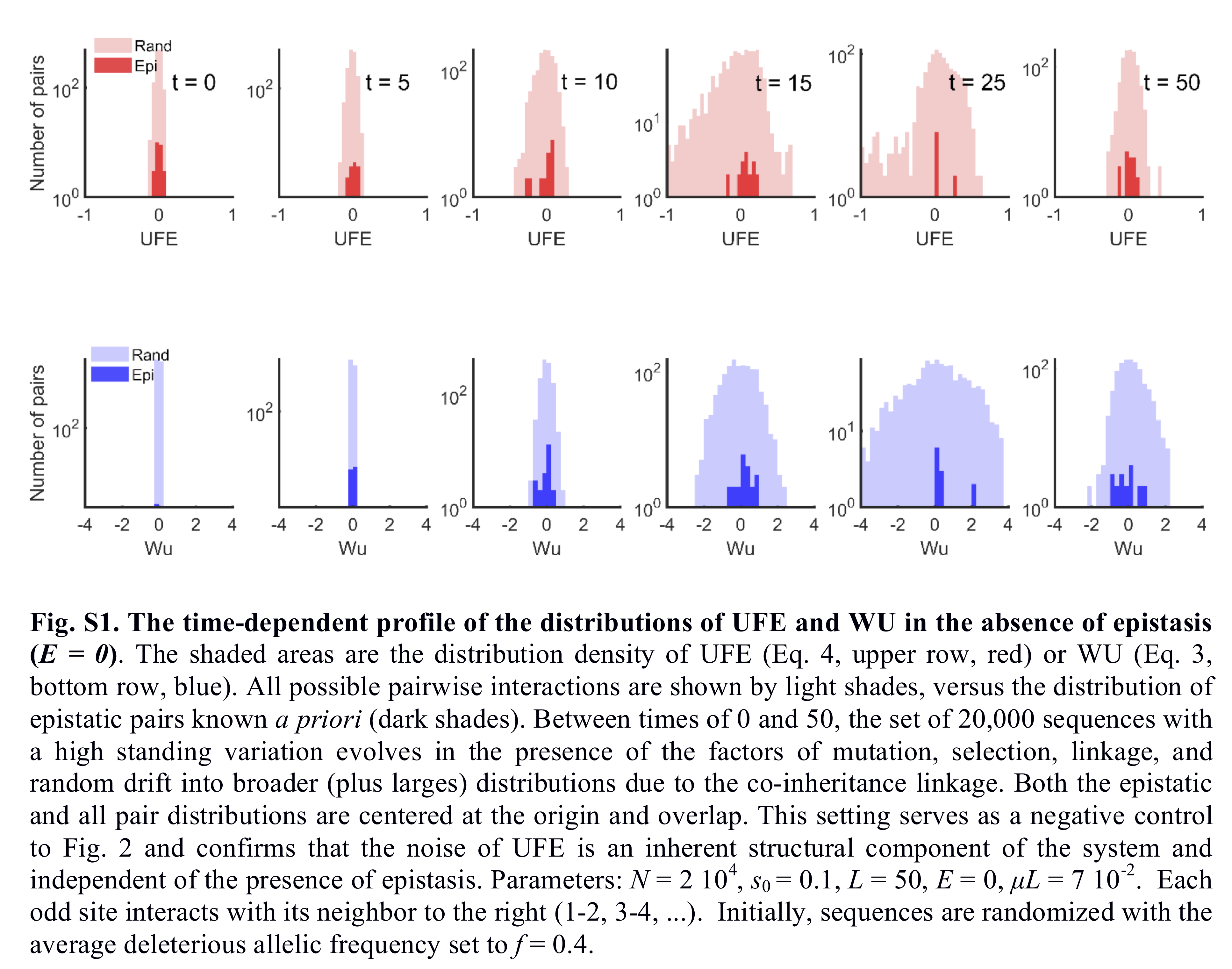

Supplement: S1 Fig — The shaded areas are the distribution density of UFE (Eq 4, upper row, red) or WU (Eq 3, bottom row, blue). All possible pairwise interactions are shown by light shades, versus the distribution of epistatic pairs known a priori (dark shades). Between times of 0 and 50, the set of 20,000 sequences with a high standing variation evolves in the presence of the factors of mutation, selection, linkage, and random drift into broader (plus larges) distributions due to the co-inheritance linkage. Both the epistatic and all pair distributions are centered at the origin and overlap. This setting serves as a negative control to Fig 2 and confirms that the noise of UFE is an inherent structural component of the system and independent of the presence of epistasis. Parameters: N = 2 104, s0 = 0.1, L = 50, E = 0, μL = 7 10−2. Each odd site interacts with its neighbor to the right (1–2, 3–4, …). Initially, sequences are randomized with the average deleterious allelic frequency set to f = 0.4. (TIFF) [file pone.0214036.s003.tiff]

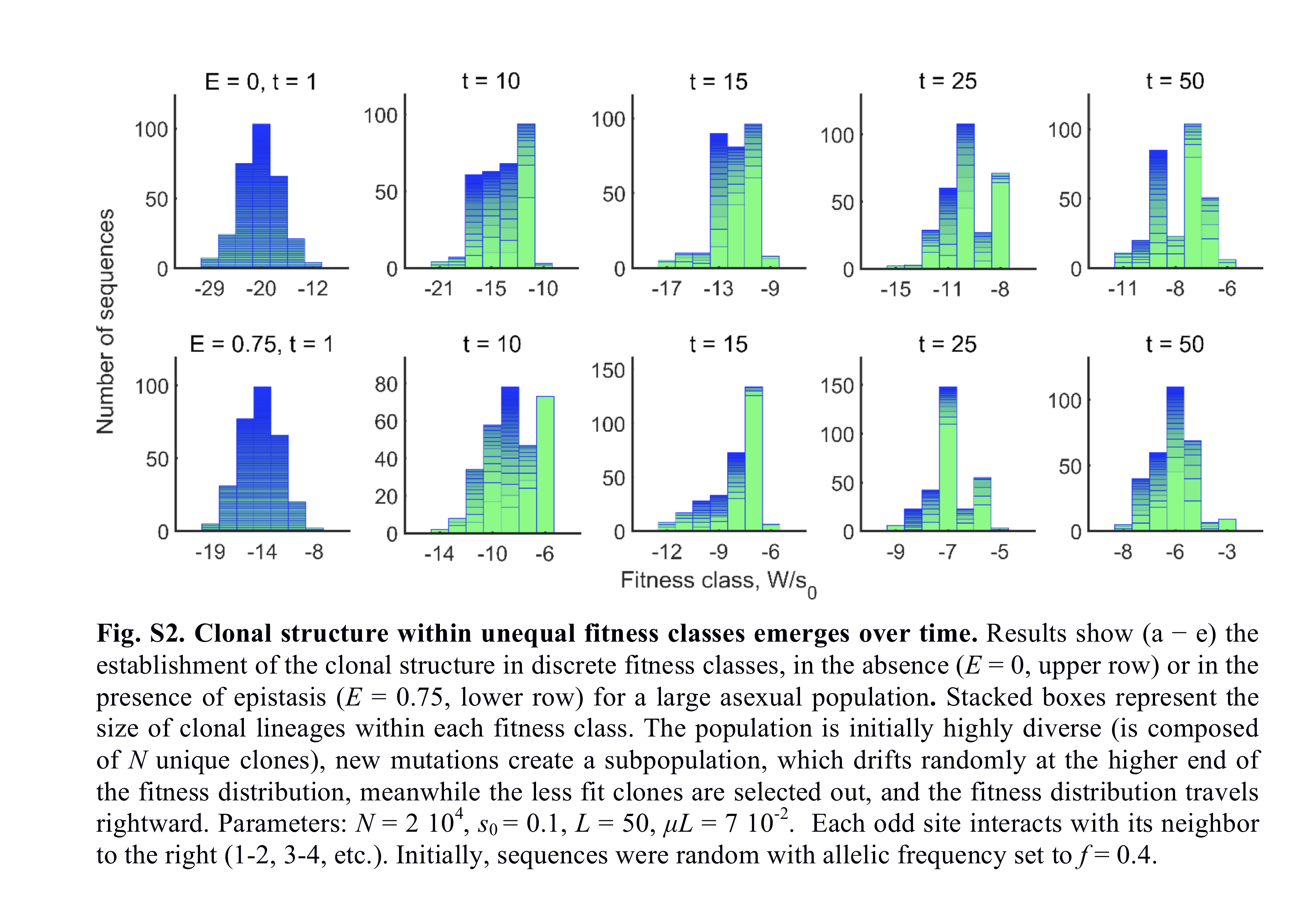

Supplement: S2 Fig — Results show (a − e) the establishment of the clonal structure in discrete fitness classes, in the absence (E = 0, upper row) or in the presence of epistasis (E = 0.75, lower row) for a large asexual population. Stacked boxes represent the size of clonal lineages within each fitness class. The population is initially highly diverse (is composed of N unique clones), new mutations create a subpopulation, which drifts randomly at the higher end of the fitness distribution, meanwhile the less fit clones are selected out, and the fitness distribution travels rightward. Parameters: N = 2 104, s0 = 0.1, L = 50, μL = 7 10−2. Each odd site interacts with its neighbor to the right (1–2, 3–4, etc.). Initially, sequences were random with allelic frequency set to f = 0.4. (TIFF) [file pone.0214036.s004.tiff]

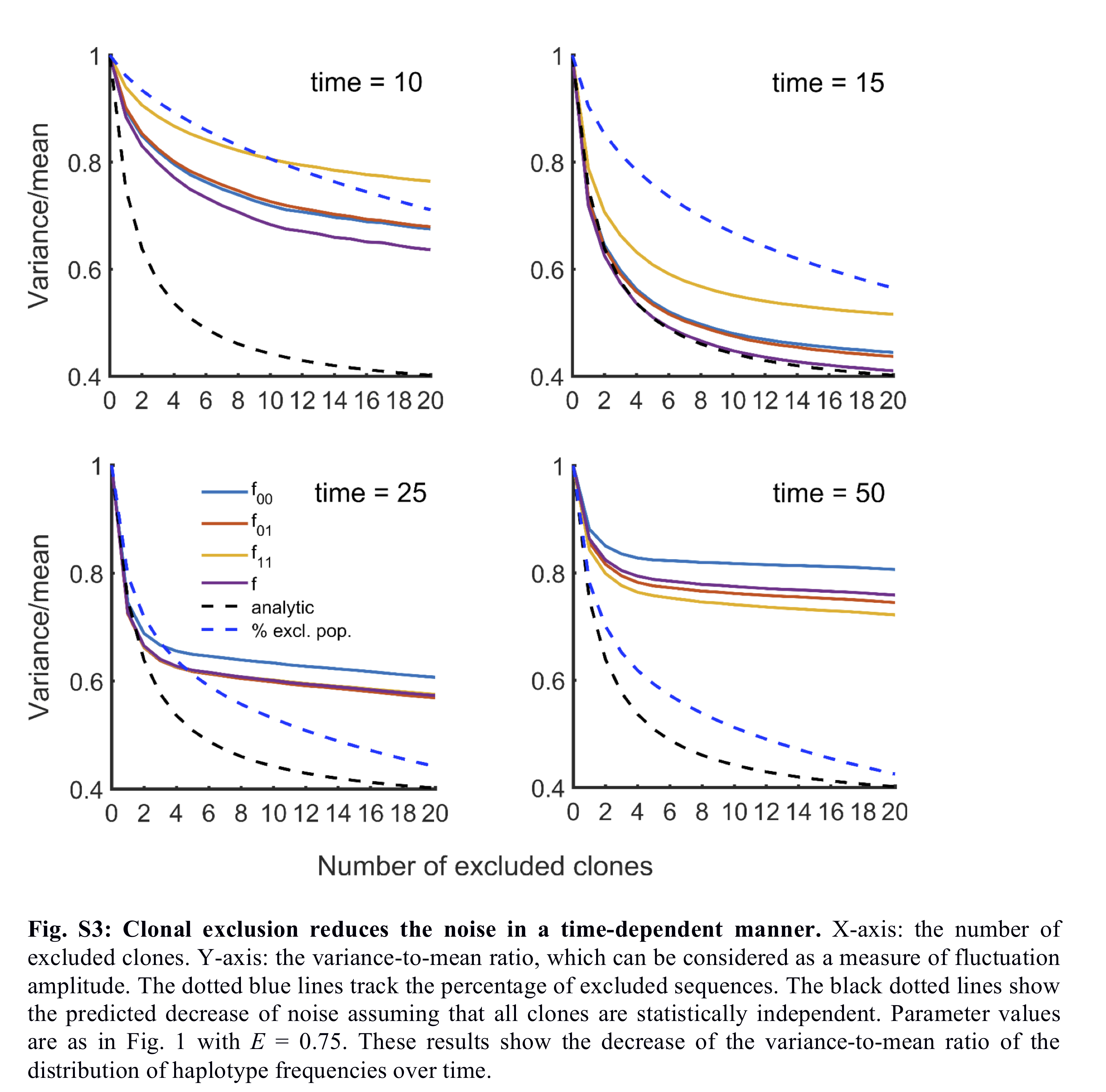

Supplement: S3 Fig — X-axis: the number of excluded clones. Y-axis: the variance-to-mean ratio, which can be considered as a measure of fluctuation amplitude. The dotted blue lines track the percentage of excluded sequences. The black dotted lines show the predicted decrease of noise assuming that all clones are statistically independent. Parameter values are as in Fig 1 with E = 0.75. These results show the decrease of the variance-to-mean ratio of the distribution of haplotype frequencies over time. (TIFF) [file pone.0214036.s005.tiff]

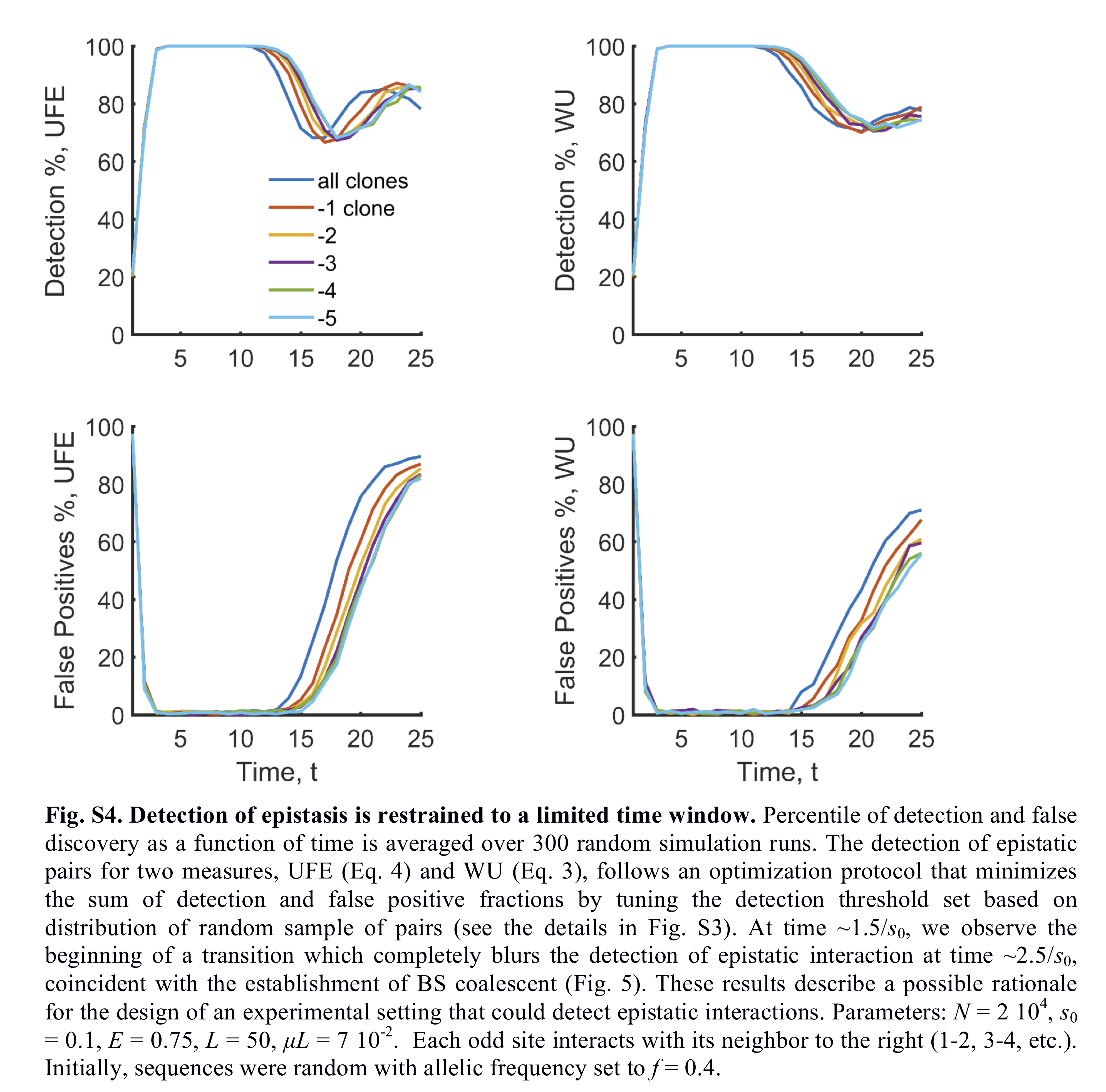

Supplement: S4 Fig — Percentile of detection and false discovery as a function of time is averaged over 300 random simulation runs. The detection of epistatic pairs for two measures, UFE (Eq 4) and WU (Eq 3), follows an optimization protocol that minimizes the sum of detection and false positive fractions by tuning the detection threshold set based on distribution of random sample of pairs (see the details in S3 Fig). At time ~1.5/s0, we observe the beginning of a transition which completely blurs the detection of epistatic interaction at time ~2.5/s0, coincident with the establishment of BS coalescent (Fig 5). These results describe a possible rationale for the design of an experimental setting that could detect epistatic interactions. Parameters: N = 2 104, s0 = 0.1, E = 0.75, L = 50, μL = 7 10−2. Each odd site interacts with its neighbor to the right (1–2, 3–4, etc.). Initially, sequences were random with allelic frequency set to f = 0.4. (TIFF) [file pone.0214036.s006.tiff]

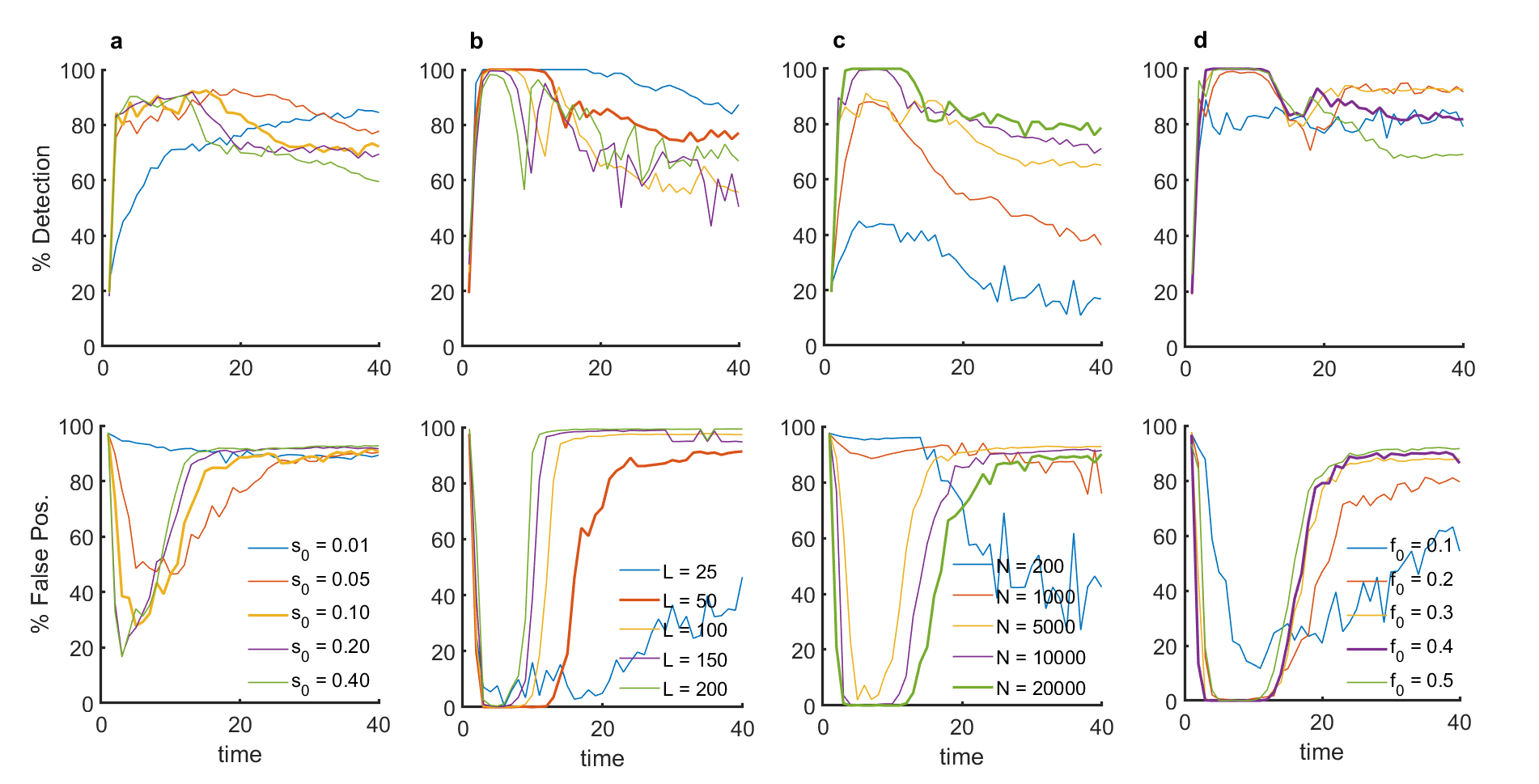

Supplement: S5 Fig — Percentile of detection and false discovery as a function of time is averaged over 25 random simulations (runs) in a broad range of parameters values. The default parameter set is E = 0.75, with the other parameters as in Fig 1. Parameters values are shown. Thick lines correspond to default parameter values used in Fig 1. (a) Distributed values of s, randomly drawn from a half-Gaussian distribution of deleterious alleles with average s0 (shown). (b) Increasing genome length L closes the detection window. (c) Decrease in population size N narrows the detection window. (d) The initial standing variation with average frequency of deleterious alleles f0 allows detection in a narrow time window at f0 > 10%. (TIF) [file pone.0214036.s007.tif]

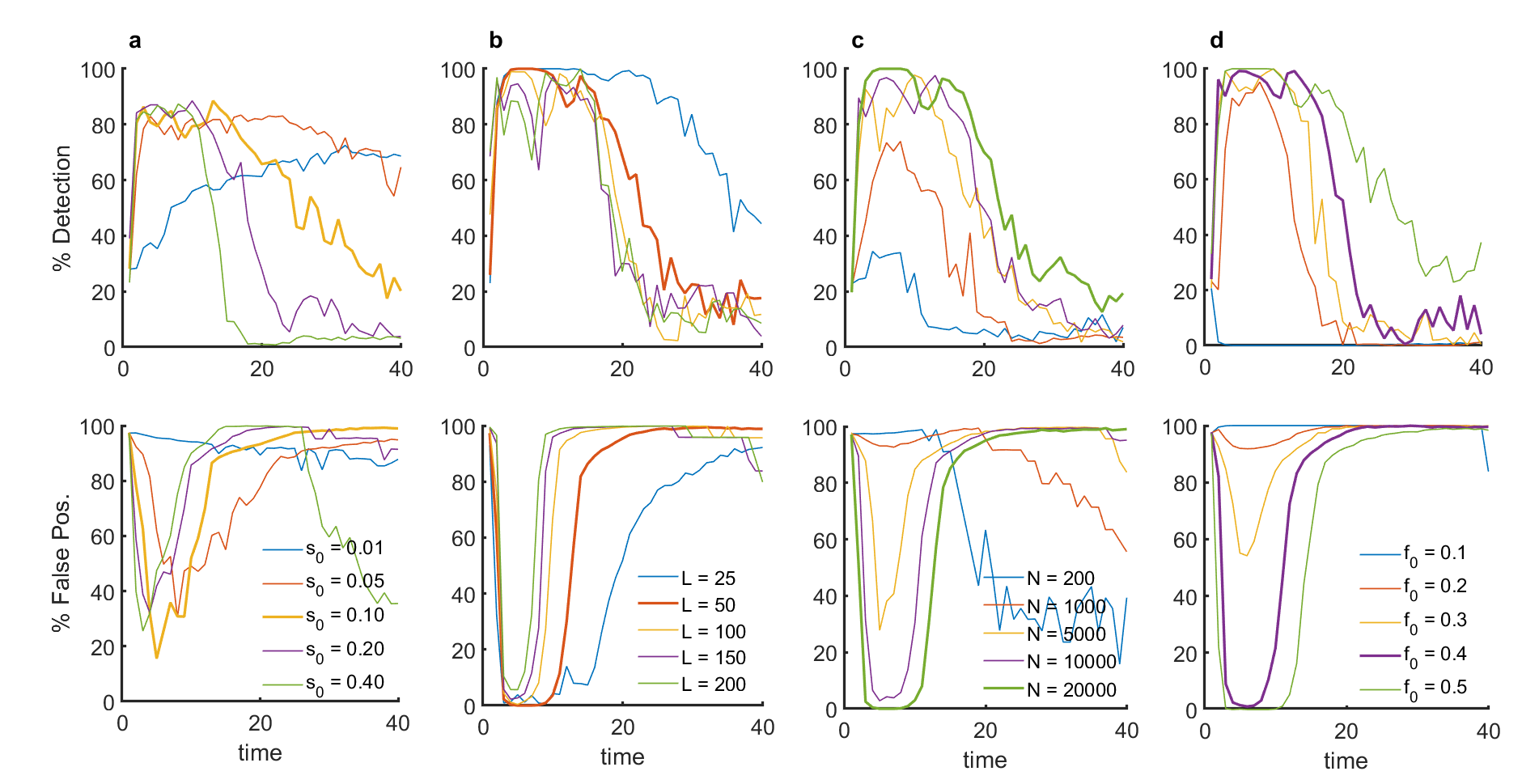

Supplement: S6 Fig — Notation as in S5 Fig. (TIF) [file pone.0214036.s008.tif]

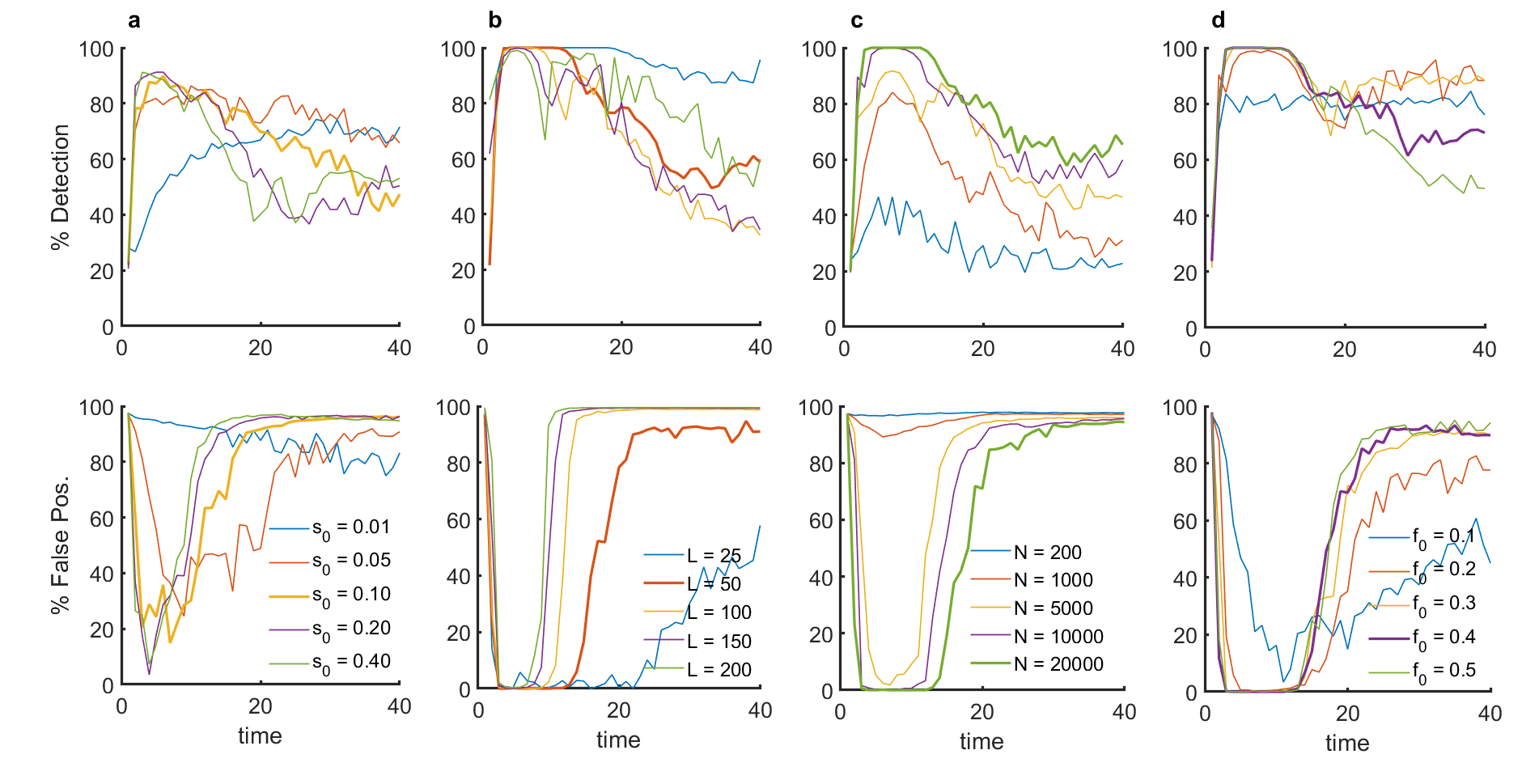

Supplement: S7 Fig — Notation as in S5 Fig. (TIF) [file pone.0214036.s009.tif]

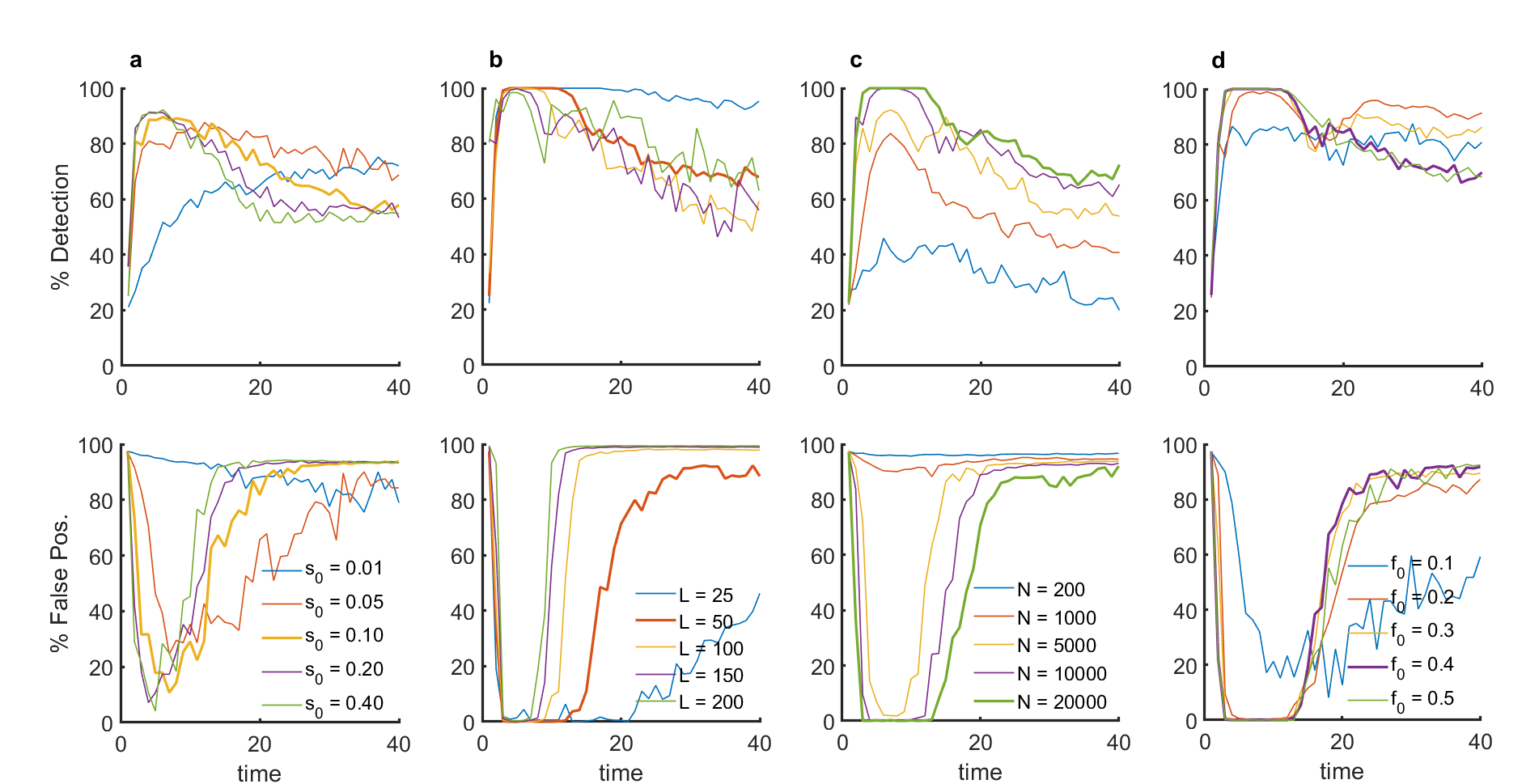

Supplement: S8 Fig — Notation as in S5 Fig. (TIF) [file pone.0214036.s010.tif]

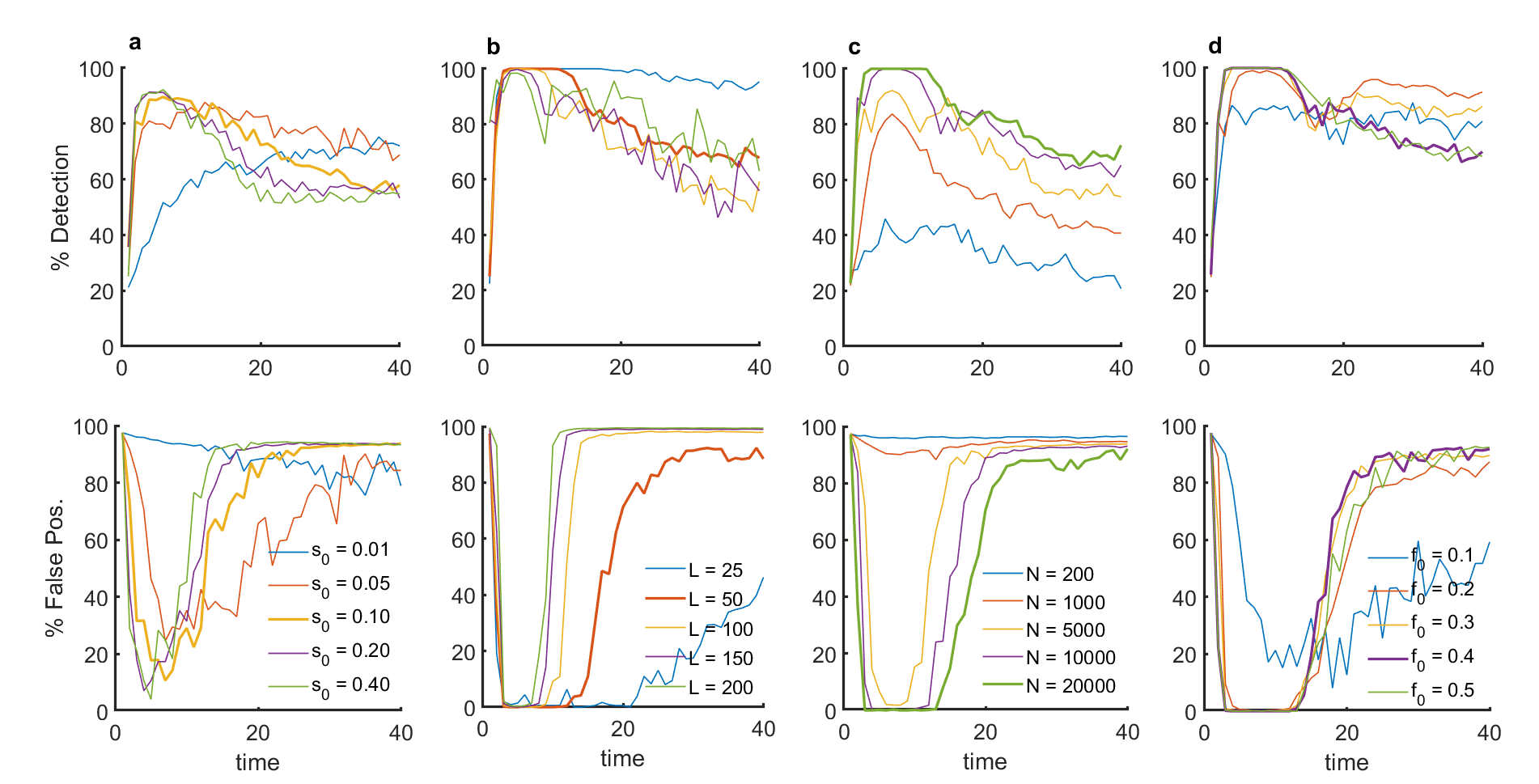

Supplement: S9 Fig — Notation as in S5 Fig. (TIF) [file pone.0214036.s011.tif]

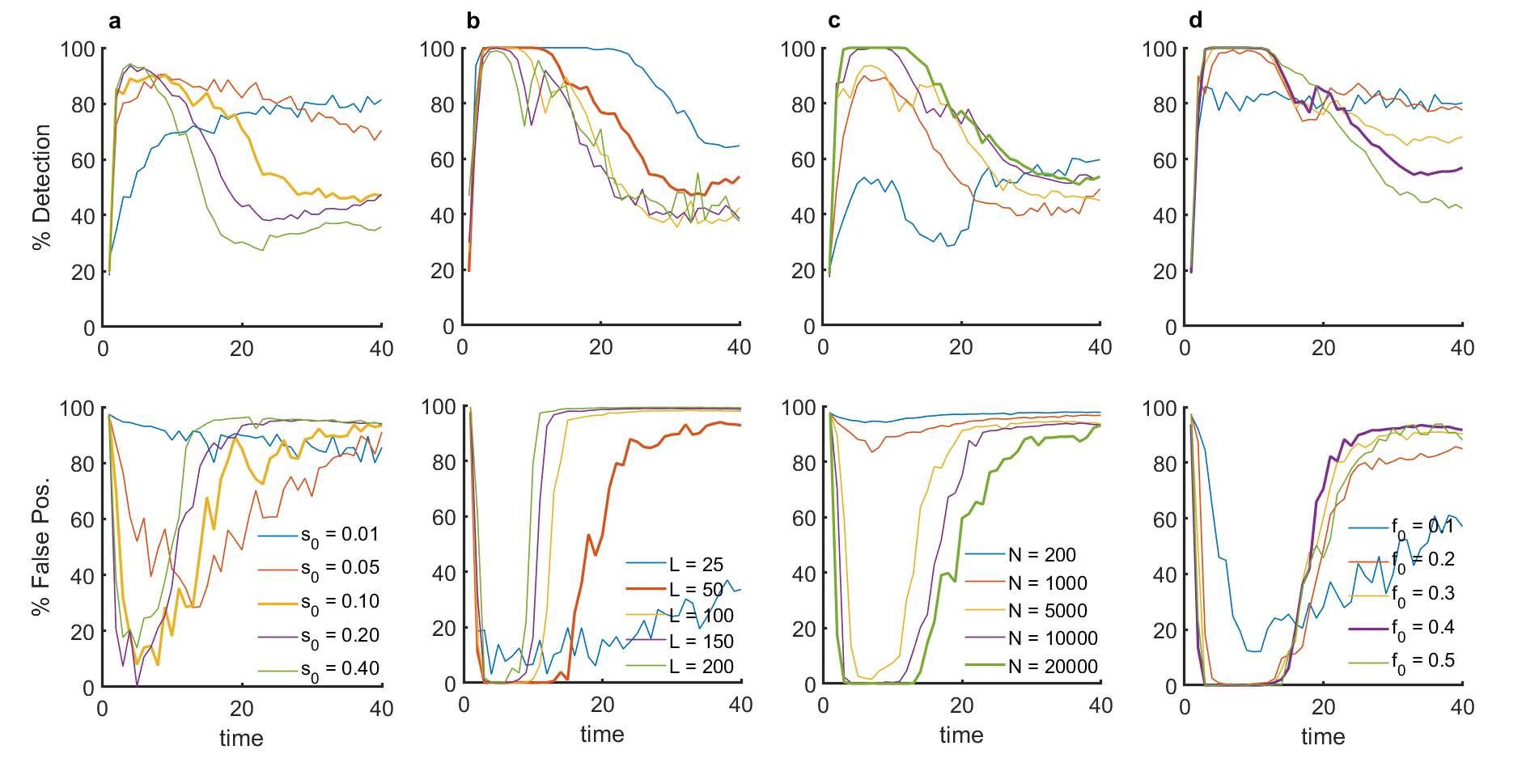

Supplement: S10 Fig — Notation as in S5 Fig. (TIF) [file pone.0214036.s012.tif]
